# Microarray Expression Profiles of 20.000 Genes across 23 Healthy Porcine Tissues

**DOI:** 10.1371/journal.pone.0001203

**Published:** 2007-11-21

**Authors:** Henrik Hornshøj, Lene Nagstrup Conley, Jakob Hedegaard, Peter Sørensen, Frank Panitz, Christian Bendixen

**Affiliations:** Department of Genetics and Biotechnology, Faculty of Agricultural Sciences, University of Aarhus, Tjele, Denmark; Deutsches Krebsforschungszentrum, Germany

## Abstract

**Background:**

Gene expression microarrays have been intensively applied to screen for genes involved in specific biological processes of interest such as diseases or responses to environmental stimuli. For mammalian species, cataloging of the global gene expression profiles in large tissue collections under normal conditions have been focusing on human and mouse genomes but is lacking for the pig genome.

**Methodology/Principal Findings:**

Here we present the results from a large-scale porcine study establishing microarray cDNA expression profiles of approximately 20.000 genes across 23 healthy tissues. As expected, a large portion of the genes show tissue specific expression in agreement with mappings to gene descriptions, Gene Ontology terms and KEGG pathways. Two-way hierarchical clustering identified expected tissue clusters in accordance with tissue type and a number of cDNA clusters having similar gene expression patterns across tissues. For one of these cDNA clusters, we demonstrate that possible tissue associated gene function can be inferred for previously uncharacterized genes based on their shared expression patterns with functionally annotated genes. We show that gene expression in common porcine tissues is similar to the expression in homologous tissues of human.

**Conclusions/Significance:**

The results from this study constitute a valuable and publicly available resource of basic gene expression profiles in normal porcine tissues and will contribute to the identification and functional annotation of porcine genes.

## Introduction

The microarray technology is ideal for whole-genome and large-scale profiling of gene expression under various conditions. For instance, microarray-based experiments have been widely used to identify differentially expressed genes involved in specific biological processes such as disease or response to environmental stimuli. These experiments have found new gene functions and provide insights into the transcriptional regulation that underlies biological processes.

Several gene expression surveys from human and mouse studies have demonstrated important applications of gene expression profiles obtained from normal functioning organs and tissues [1]–[8]. For example, genes that are tissue specific have been identified and used to describe the biological processes associated with human organs [9]. In addition, integration of gene expression profiles from healthy tissues has been shown to be valuable in the biological interpretation of expression profiles from human cancer cells [10]. Compendiums of gene expression, such as Human Gene Expression (HuGE) Index [11], Gene Normal Tissue Expression (GeneNote) [12] and SymAtlas [13], have also been created as publicly available web resource as a result of gene expression surveys. They provide easy access to tissue expression levels for single genes. Finally, co-expressed genes have been used to predict function of previously uncharacterized genes [5].

Domesticated pig (Sus scrofa) was our choice of model organism for large scale gene expression profiling as it provides easy access to samples of tissues, which are physiologically and anatomically similar to those of other mammalians used in biomedical research, such as human and mouse. At present, no porcine whole genome surveys of gene expression across large tissue collections have been reported. Here we present the results from a large-scale porcine survey of expression profiles of 26.877 microarray cDNAs representing approximately 20.000 genes in 23 healthy tissues. The overall intention of this study has been to catalog the basic expression profiles of as many genes as possible in a large collection of normal functioning porcine tissues and make this publicly available. The results from this study have been made publicly available via Gene Expression Omnibus (GEO) [14].

Gene annotations such as Gene Ontology (GO) terms and KEGG pathways are integrated in the analysis and interpretation of expression profiles. GO terms constitute a controlled vocabulary of Biological Processes (BP), Molecular Functions (MF) and Cellular Components (CC) for gene products [15]. GO has been widely used as a tool for the interpretation of microarray differential gene expression by grouping genes according to mapped GO terms instead of looking at single genes. One common approach is to statistically test for enriched ontology terms in microarray data [16]. The Kyoto Encyclopedia of Genes and Genomes (KEGG) represent the current knowledge on molecular interaction networks such as pathways [17]. By KEGG classification of microarray genes one can identify pathways that are associated with certain phenotypes or disease states, for example leukemia [18].

We identify single genes with differences in expression across tissues and use two-way hierarchical clustering to group tissues and genes according to their expression profiles. In an attempt to infer potential tissue functions for previously uncharacterized genes, we investigate the expression patterns, functional annotations and cDNA sequences of a gene cluster. Finally, we compare the gene expression profiles of orthologous genes in nine common tissues across pig and human.

## Results

### Establishing microarray gene expression profiles

In total, 46 two-channel hybridizations were carried out corresponding to 23 tissue samples each with two independent RNA extractions. A common reference sample was constructed from a pool of all labeled RNA extracts. The ratio between the mRNA levels in each tissue and the common reference was computed for all microarray cDNA spots generating 46 sets of relative gene expression profiles for 26.877 porcine cDNAs (PCs). The data was normalized using the print tip loess method as described in the methods section. The raw intensities and normalized gene expression ratios were made publicly available by submission to GEO and can be accessed via accession number GSE4918.

### Assigning known processes and pathways to tissue gene expression

**Figure 1:**
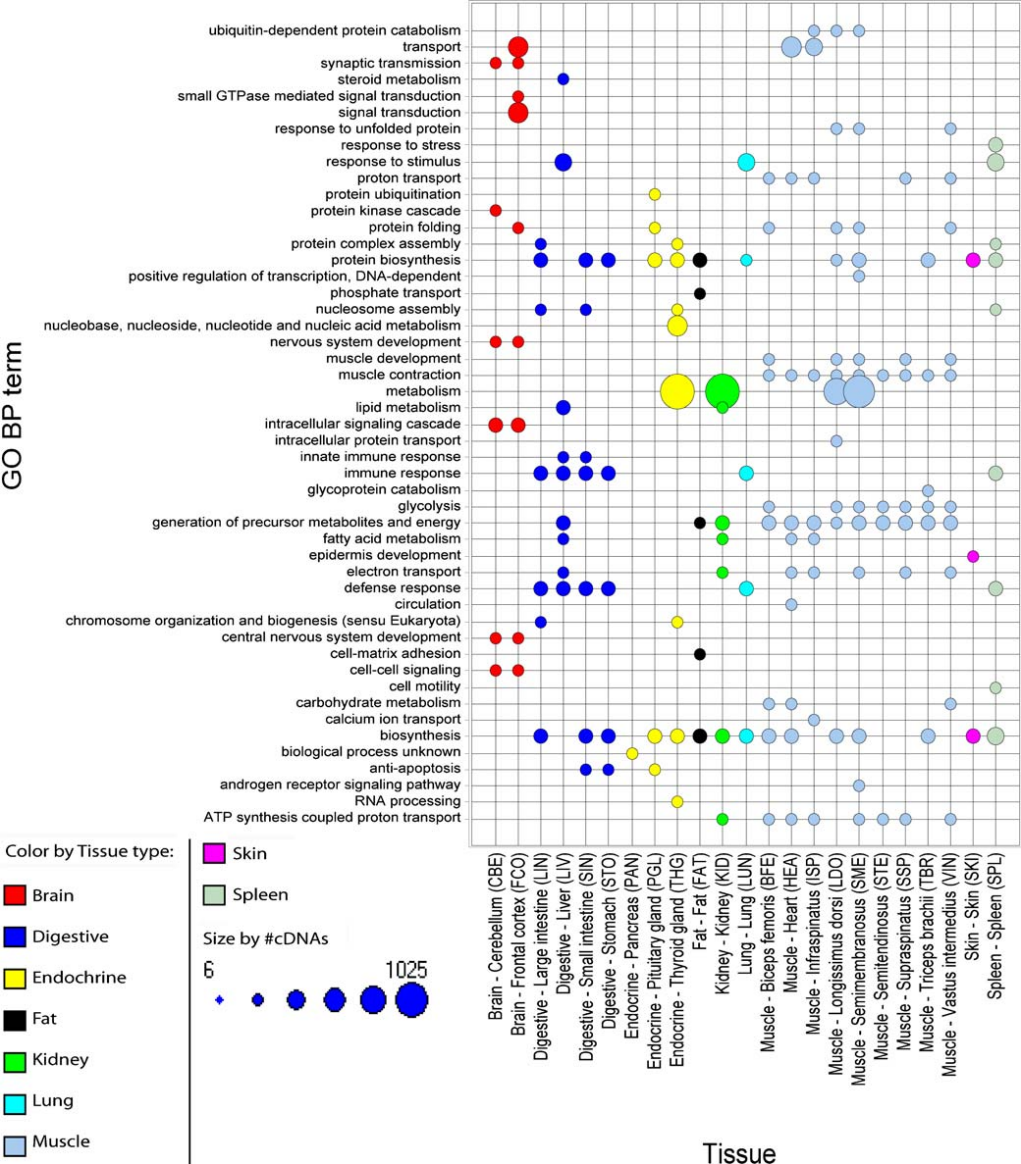
Enriched GO BP terms for cDNAs representing positively regulated genes. Size of dots corresponds to the number of cDNAs that were tested (minimum = 6 and maximum = 1025) and color codes indicate tissue type. Only significant (P≤0.01) GO-BP terms represented by 50 or more cDNAs on the array were included.

We first identified differences between the expression in each of the 23 tissues and the overall expression defined by the expression in other 22 tissues. The microarray cDNAs were mapped to GO terms of class BP to assign known biological processes to the identified tissue differences in expression. The GO terms mapped to positively regulated genes were subjected to an enrichment test to compare tissue expression with known biological processes (Figure 1). As expected, we were able to find numerous expected matches between observed tissue specific gene expression and GO biological processes that directly associate with specific tissues. For the genes showing increased expression in muscle type tissues we found ‘muscle contraction’ to be a significantly enriched GO BP term in all of the nine muscle type tissues used in the experiment. Similarly ‘muscle development’ was found to be an enriched GO BP term in five muscle tissues (Biceps femoris, Longissimus dorsi, Semimembranosus, Supraspinatus and Vastus intermedius). Increased expression of genes assigned to ‘circulation’ was observed exclusively in heart tissue. Longissimus dorsi and Semimembranosus appear to be the most similar tissues among the muscle type tissues in terms of sharing most GO BP terms (10 out of 49). Genes assigned to the liver associated process ‘steroid metabolism’ are positively regulated in the liver tissue. Genes assigned to brain processes such as ‘synaptic transmission’, ‘nervous system development’ and ‘intracellular signaling’ were found to have specifically higher expression levels in Cerebellum and Frontal cortex. ‘Epidermis development’, a process associated with skin, is assigned to genes showing increased expression levels in skin. ‘Biosynthesis’ is the most frequent process associated with increased gene expression by its appearance in 15 of the 23 tissues.

In general, none of the tissue-specific terms were found to be enriched for genes positively regulated in other tissues than expected. However, some tissue-specific terms were not enriched in all those tissues as might have been expected. For example the process ‘muscle development’ was only found for positively regulated genes in five of the nine muscle tissues.

**Figure 2:**
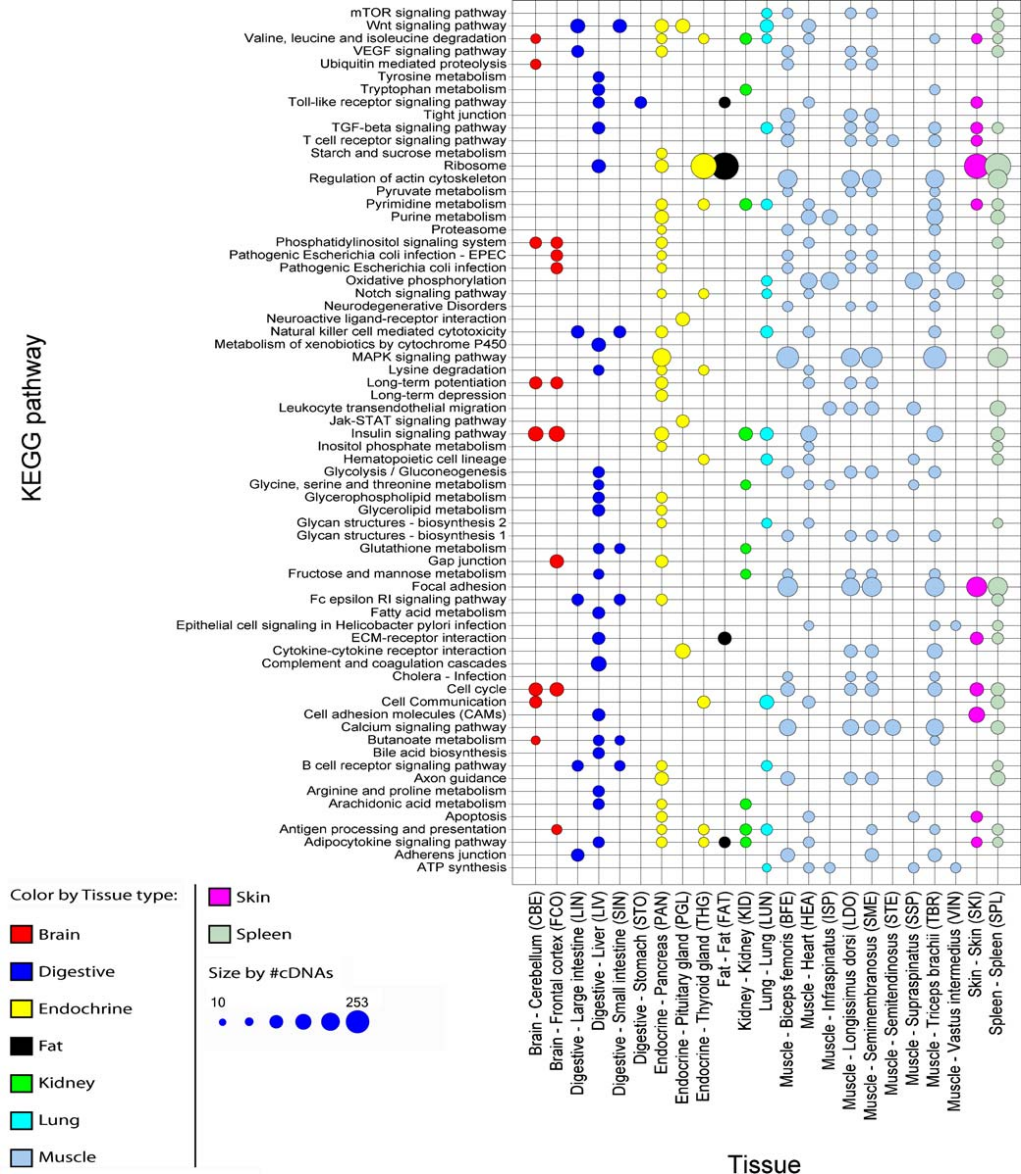
KEGG pathways for cDNAs representing differently expressed genes across tissues. Size of dots corresponds to the number of cDNAs (minimum = 10 and maximum = 253) that were tested to have a positive influence on the expression levels and color codes indicate tissue type. Only significant (P≤0.05) KEGG pathways represented by 50 or more cDNAs on the array were included.

The genes represented on the microarray where also mapped to available KEGG pathways based on the gene accession IDs. The cDNAs were grouped by these pathways and a global test was carried out on the normalized data set to assign possible biochemical pathways with positively regulated genes (Figure 2). For several pathway genes we identified expected tissue specific expression profiles in clear agreement with the pathway function. For example, ‘regulation of actin cytoskeleton’ is a muscle associated pathway and the genes that represent this pathway on the microarray show increased expression in four muscle type tissues (Biceps femoris, Longissimus dorsi, Semimembranosus and Triceps brachii). Again, Longissimus dorsi and Semimembranosus appear to be the most similar tissues among the muscle type tissues. These two tissues share 24 out of 68 KEGG pathways whose genes are positively regulated. Two known liver tissue associated pathways named ‘metabolism of xenobiotics by cytochrome P450’ and ‘complement and coagulation cascades’ are represented by genes that are positively regulated in the liver tissue.

### Clustering of gene expression profiles by tissues

**Figure 3:**
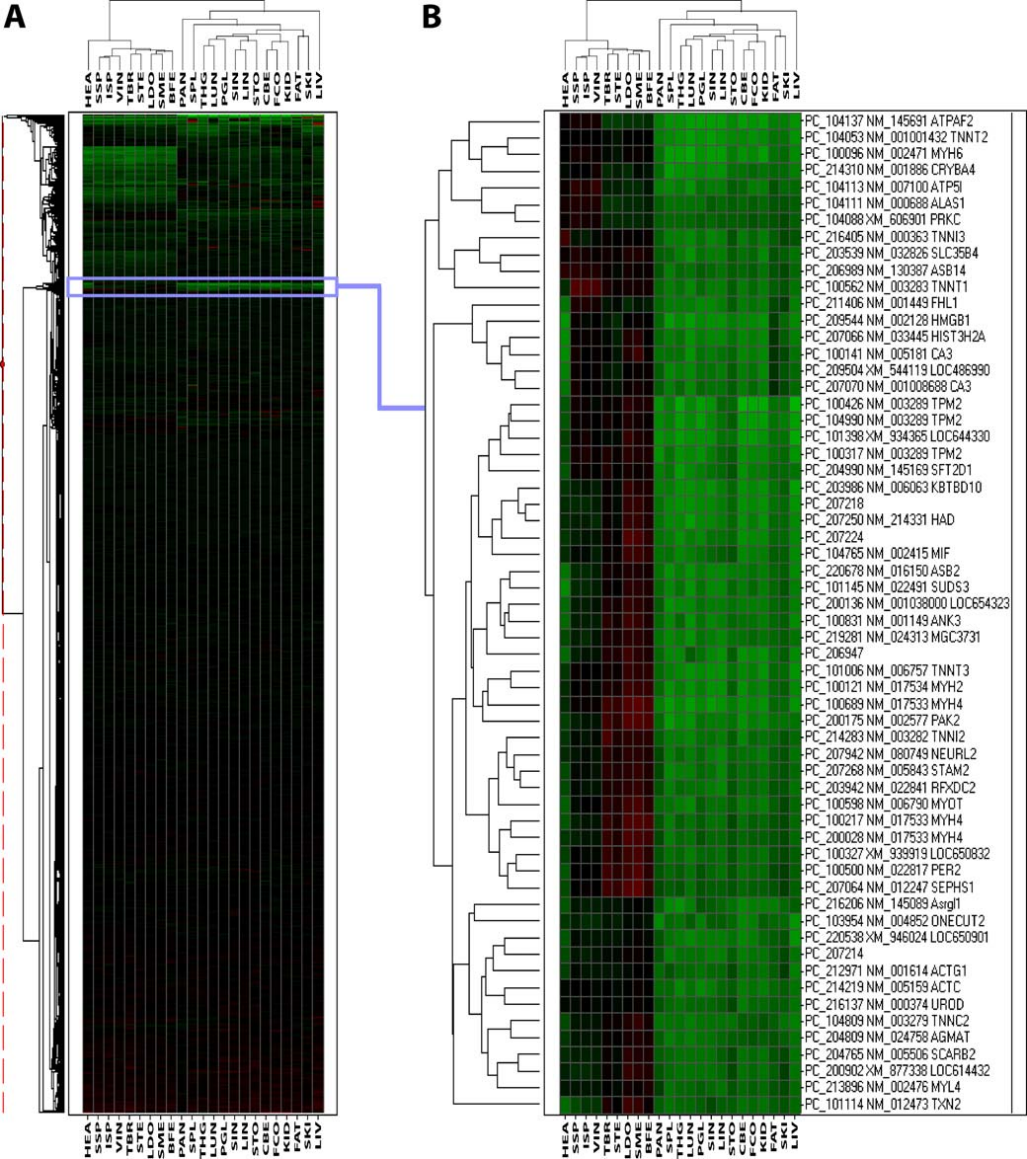
Two-way hierarchical clustering of gene expression ratios. Heatmaps displays the log2(M) on a color scale from green indicating lower expression to red indicating a higher expression, interpolated over black for log2(M) = 0. A. Overview of entire hierarchical clustering showing 26.877 cDNAs (row-wise) and 23 tissues (column-wise). B. Enlarged view of selected cDNA cluster. BFE, Biceps femoris; CBE, Cerebellum; FAT, fat; FCO, Frontal cortex; HEA, heart; ISP, Infraspinatus; KID, kidney; LDO, Longissimus dorsi; LIN, large intestine; LIV, liver; LUN, lung; PAN, pancreas; PGL, pituitary gland; SIN, small intestine; SKI, skin; SME, Semimembranosus; SPL, spleen; SSP, Supraspinatus; STE, Semitendinosus; STO, stomach; TBR, Triceps brachii; THG, thyroid gland; VIN, Vastus intermedius.

Two-way hierarchical clustering was applied to the normalized expression profiles to identify clusters of genes and tissues displaying similar expression patterns (Figure 3A). The tissue dendogram shows that the tissues cluster into groups as expected from their tissue types. The nine muscle type tissues Biceps femoris, heart, Infraspinatus, Longissimus dorsi, Semimembranosus, Supraspinatus, Semitendinosus, Triceps brachii and Vastus intermedius cluster into one single tissue group. Although located in this muscle cluster, the expression profiles of heart tissue is slightly different than those of the other muscle tissues. Infraspinatus and Supraspinatus, which are both muscles on the dorsum of the scapula, form a subcluster in the muscle tissue group. Thyroid gland and pituitary gland are members of the same cluster, which surprisingly also includes lung. The fact that thyroid gland and lung tend to cluster together may result from shared positively regulated genes involved in amino acid metabolisms and cell adhesion (see Figure 2). Large intenstine, small intestine and stomach form a group of digestive tissues of which the first two are located in a separate subcluster. Likewise, Frontal cortex and Cerebellum, define a cluster of brain tissues. Pancreas and liver tissues show very distinct expression patterns compared to any other tissues.

### Co-expression of uncharacterized genes with known genes

Several cDNA clusters, whose members share expression profiles across tissues, can be identified on top part of the heatmap in Figure 3A. One of the identified cDNA clusters has 60 members of which most are positively regulated in muscle type tissues and negatively regulated in non-muscle type tissues (see Figure 3B). We selected this cDNA cluster for further investigation in an attempt to infer functions for uncharacterized genes based on co-expression with known genes and sequence analysis. As anticipated, the description for the genes mapped to these cluster cDNAs reveals many genes with muscle-specific function, for example myosins (MYH2, MYH4, MYH6 and MYL4), tropomyosin (TPM2), myotilin (MYOT), troponins (TNNC2, TNNI2, TNNI3, TNNT1, TNNT2 and TNNT3), carbonic anhydrase III (CA3) and actins (ACTC and ACTG1). Thus, the observed muscle specific expression profiles of this cluster are largely confirmed by the assigned gene annotations.

**Figure 4:**
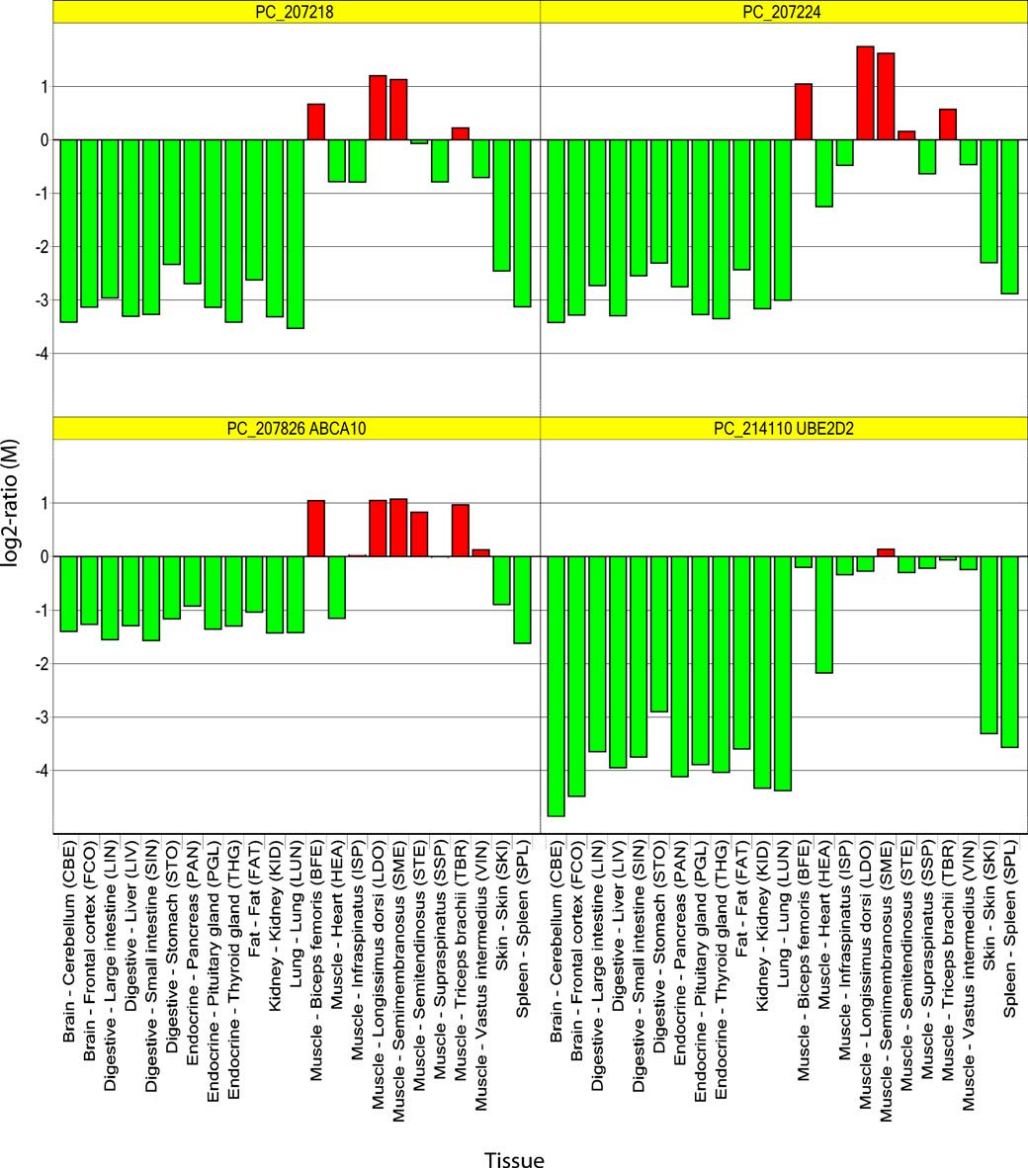
Expression profiles of uncharacterized genes and co-expressed genes with known function. Each histogram represents the expression profiles of a single cDNA across all 23 tissues for two uncharacterized protein coding genes (PC_207218, PC_207224) and four co-expressed genes with known function (PC_207826: ABCA10, PC_214110: UBE2D2). Each bar represents the gene expression ratio (M) between the tissue sample and the common reference sample on the logarithmic scale. M values below zero, indicating lower gene expression level, are shown by green bars. M values above zero, indicating higher expression, are shown by red bars.

There are four non-annotated cDNA members (PC_206947, PC_207214, PC_207218, PC_207224) of this particular cluster (see Figure 3B). The sequences of these cDNA members do not show significant nucleotide BLASTN similarity (P-value≤0.1) to any known human or other mammalian gene transcript. These cDNAs were considered to represent porcine genes with no available annotations, but whose expression is predominantly muscle specific. We further investigated the cDNA sequences and gene expression patterns of this cluster to infer the possible function of the corresponding uncharacterized genes. We first compared the cDNA sequences with publicly available porcine genome sequences at Ensembl Trace Server [19] to increase the sequence representation and found genome sequence(s) with similarity to three of the cDNAs (PC_207214, PC_207218, PC_207224). A BLASTP comparison of the amino acid translated genome sequences to NCBI's non-redundant (nr) sequence database [20] identified similarity (P-value = 5×10^−37^) to an additional protein for PC_207218 termed ATP-binding cassette transporter G1 (ABCG1) from human. Another cDNA (PC_207826) is found on the microarray whose gene also encodes an ABC transporter protein termed ATP-binding cassette, sub-family A (ABC1), member 10 (ABCA10). Also, the expression patterns of these two ABC transporter genes represented by PC_207218 and PC_207826 are similar (see Figure 4). Protein domain prediction in the amino acid sequence translated from cDNA and genome sequences using the SMART web program [21] showed the presence of transmembrane regions in the protein products of the assumed genes represented by PC_206947, PC_207214, PC_207218. Signal peptides were also found in the amino acid sequences of PC_207214 and PC_207218. The amino acid sequence for PC_207224 was predicted to contain an ubiquitin conjugating-like (UBC-like) protein domain (P-value = 1.7×10^−2^). Another microarray cDNA named PC_214110 represents the gene encoding ubiquitin-conjugating enzyme E2D 2 (UBE2D2) and the expression patterns if this gene appears to be similar to PC_207224 (see Figure 4).

### Correlation with gene expression in human tissues

To identify similarities in tissue gene expression between pig and human we computed Pearson's correlation coefficients for pairs of common tissues across these two species. We first identified a study, in which the gene expression profiles of 35 human tissue types were also established using a cDNA-based microarray experiment with a common reference design [7]. Nine common tissues were found to be present in both our study and the human study (Frontal cortex, heart, kidney, liver, lung, pancreas, spleen, stomach and Thyroid gland). We identified orthologous relationship for 3.861 genes across the microarray platforms of these two studies based on the best reciprocal BLASTN hit between cDNA sequences and identical mapping of cDNA sequences to gene IDs (see Methods). We then extracted the expression data for the 3.861 orthologous genes in the nine common tissues of these two studies. In order to make the gene expression profiles from these two studies as comparable as possible we followed a recently proposed approach, in which relative abundance (RA) values are computed as the measurement for gene expression levels and used for cross-species comparison [22]. RA is defined by the raw signal intensities in the individual tissues divided by the sum of raw signal intensity in all nine tissues.

**Figure 5:**
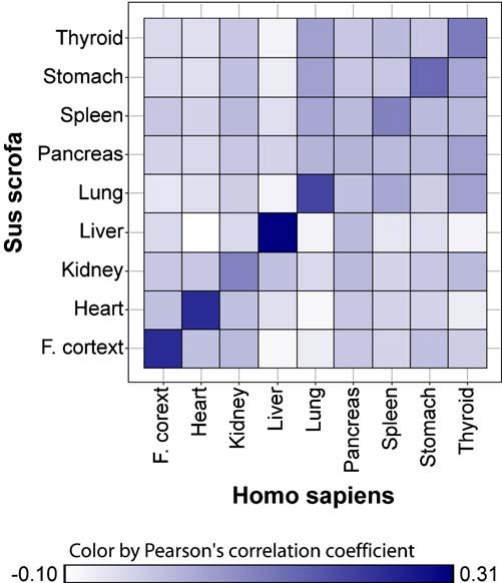
Correlation in gene expression between common tissues of pig and human. Pearson's correlation coefficients computed for pairs of common tissues from pig and human. Color scale from white to dark blue represents correlation coefficients from −0.10 to 0.31.

Using Pearson's method we computed pair wise correlation coefficients between common tissues across pig and human, which is shown in Figure 5. The correlation in gene expression between porcine and human is clearly higher for homologous tissue pairs in comparison to the correlation between non-homologous tissues with the exception of pancreas. The highest correlation was found for liver followed by Frontal cortex, heart, lung, stomach, thyroid, spleen and kidney.

## Discussion

We carried out a large-scale survey of gene expression in porcine by establishing expression profiles for approximately 20.000 genes represented by 26.877 microarray cDNAs across 23 healthy and diverse tissue samples. We analyzed the expression in each tissue compared the overall expression.

Increased tissue specific gene expression corresponds to numerous of the major tissue specific GO BP terms and KEGG pathways as expected (see Figure 1 and 2). The muscle type tissues Longissimus dorsi and Semimembranosus share most enriched GO terms and KEGG pathways among the muscle type tissues. Longissimus dorsi is one of the deep muscles of the back whereas Semimembranosus is situated at the back of the thigh, but both muscles are type II muscle fibers. The integration of functional gene annotations in the analysis confirms the detected differences in gene expression across tissues and confirms the expression of porcine genes being as expected in terms of assigned known tissue associated function.

Two-way hierarchical clustering of the gene expression profiles formed expected clusters according to tissue types (see Figure 3). The gland tissue thyroid gland and pituitary gland are both members of the same cluster. Thyroid gland forms a subcluster together with lung. This might be explained from the fact that these two tissues share positive regulation of genes that are mapped to the KEGG pathways of several amino acid metabolisms and cell adhesion molecules (CAMs) (see Figure 2).

Clusters of cDNAs who share expression profiles across one or more tissues were identified by two-way hierarchical clustering. In an attempt to identify expression of uncharacterized genes and infer possible function for these genes we selected one of these clusters and investigated the shared expression patterns with known genes and cDNA sequences. This cluster has 60 cDNA members predominantly with high expression levels in muscle type tissues and low expression levels in non-muscle type tissues (see Figure 3).

Four members of the selected cDNA cluster did not produce significant BLASTN nucleotide similarity to known human protein coding sequences in NCBI's nr sequence database. These cDNAs were considered to represent previously uncharacterized genes. A further analysis of expression profiles and sequence analysis was carried out on the cluster cDNAs in order to infer potential function for these muscle specific genes. The fact that protein domains can be predicted in the translated amino acid sequences suggest that the cDNA sequences are in fact protein coding and the missing sequence similarity to other protein coding sequences suggest the existence of novel and expressed porcine genes. These assumed novel genes encode proteins that are likely involved in muscle-specific processes such as ‘muscle development’ and ‘muscle contraction’, which are frequent GO terms in this cluster. A possible reason for the missing transmembrane domain and signal peptide protein domain predictions for PC_207224 could be that it represents an untranslated region (UTR) rather than protein coding regions. An additional gene product from human, termed ATP-binding cassette transport G1 (ABCG1) was found to be similar to the translated genome sequences for the PC207218 cDNA. The ABCG1 gene is expressed in many human tissues [23], but apparently not in a muscle specific manner. The presence of an ABCG1 gene is supported by the predicted transmembrane domain in the translated amino acid sequence of PC_207218. Also the cDNA termed PC_207826 represents a member of the ABC transporter protein family named ATP-binding cassette, sub-family A (ABC1), member 10 (ABCA10), which shows similar expression across the tissues (see Figure 4). This gene has no mappings to GO terms, which could have indicated a tissue related function. The expression profiles observed in this study, however, suggest that ABC transporter proteins play a role in a muscle associated process.

In the amino acid sequence translated from the PC_207224 cDNA sequence, the SMART program predicts an ubiquitin conjugating-like (UBC-like) protein domain, although with low significance (P-value = 0.017). Increased expression of the ubiquitin - proteasome proteolytic pathway, which includes ubiquitin conjugating enzymes (E2s), has been associated with loss of muscle protein [24]. Assuming that the gene represented by PC_207224 encodes an E2 enzyme, one could speculate that this enzyme is part of a pathway associated with a process in muscle involving protein loss. Another E2 gene, termed UBE2D2, is also present on the microarray represented by PC_214110, which shows a similar expression pattern (see Figure 4).

The presence of predicted protein domains in the translated amino acid sequence suggest that the corresponding genes are novel because the cDNA sequences are predicted to be protein coding and not UTR sequence, which otherwise might have explained the missing similarity to other protein coding sequences. The increased expression patterns of these assumed novel genes in muscle type tissues suggest that the gene functions are related to muscle-specific processes. The combination of sequence analysis with gene expression profiles provides important clues to the function of uncharacterized genes and this approach makes it worthwhile to analyze the other cDNA clusters identified. Full-length cDNA cloning and sequencing should also be applied to cDNAs representing uncharacterized genes as part of the further annotation of these genes.

We have compared gene expression profiles for nine common tissues across pig and human using 3.861 orthologous genes by means of computing Pearson's correlation coefficients. We have found clear similarity between homologous tissues across these two species as would be expected. The highest similarity was observed between pig and human liver followed by the other eight tissues Frontal cortex, heart, lung, stomach, thyroid, spleen, kidney and pancreas. The fact that cross-species correlation in gene expression is higher between homologous tissue-pairs compared to the correlation between non-homologous tissue-pairs further confirms the established expression profiles of this study, since similar processes and therefore similar gene expression are expected to occur in the same common tissues of these two mammalian species. However, differences in technical aspects of the compared tissue expression studies are likely to reduce correlations and therefore the true biological tissue correlations are probably higher than the correlations obtained here.

## Materials and Methods

### Tissue samples and RNA extractions

Each of the 23 tissue samples was prepared from five healthy Hampshire gilts at age four to six months. We have used tissue sample pooling of the five gilts, a cost-effective approach for reducing effects from individuals [25] and identifying the most common differences in gene expression [26]. The tissue samples were immediately frozen in liquid nitrogen and subsequently kept at −80°C. Two independent RNA extractions (46 in total) were carried out from each tissue sample using the RNeasy Maxi Kit from Qiagen. The 23 tissues and their abbreviations used in this experiment: BFE, Biceps femoris; CBE, Cerebellum; FAT, fat; FCO, Frontal cortex; HEA, heart; ISP, Infraspinatus; KID, kidney; LDO, Longissimus dorsi; LIN, large intestine; LIV, liver; LUN, lung; PAN, pancreas; PGL, pituitary gland; SIN, small intestine; SKI, skin; SME, Semimembranosus; SPL, spleen; SSP, Supraspinatus; STE, Semitendinosus; STO, stomach; TBR, Triceps brachii; THG, thyroid gland; VIN, Vastus intermedius.

### Microarray cDNAs

The DNA fragments were amplified from cDNA clones generated as part of large-scale sequencing of expressed sequence tags (ESTs) in the Sino-Danish Pig Genome Sequencing Project [27]. The cDNA clones for PCR amplification were selected such that the cDNAs covered the largest possible number of human gene transcripts. EST clusters for human gene transcripts in NCBI's RefSeq database release 17 [28] were created using BLASTN sequence similarity program implemented to run on a DeCypher computer [http://www.timelogic.com] with P-value at or below 10^−8^. Within each cluster one cDNA with the minimum predicted distance to the 3′ end of the human gene transcript was selected. Microarray cDNAs were mapped to GO terms and KEGG pathways based on the human accession id, obtained from BLASTN, using the AnnBuilder package [29] from Bioconductor [30]. To represent uncharacterized genes on the microarray, a set of EST sequence clusters without BLASTN sequence similarity to any known human gene transcript was created and clustered using the “all-vs-all” TERACLU algorithm on a DeCypher computer from TimeLogic [http://www.timelogic.com]. We added one cDNA to the selection list for each of EST clusters with a minimum depth of 3 ESTs and minimum predicted distance to the 3′ end of the assembled EST contig. A total list of 27.744 elements consisting of 26.877 cDNAs and 867 control elements was created for spotting. Of the 26.877 cDNAs, 21.417 map to 15.831 human gene transcript IDs corresponding to roughly 1.35 cDNAs per gene transcript. The remaining 5.460 cDNAs were thus estimated to cover around 4.036 gene transcripts yielding approximately 19.867 gene transcripts in total. All elements were spotted in duplicates on UltraGAPS slides from Corning with an SDDC-2 ChipWriter (Biorad) yielding a total of 55.488 spots on each microarray. A full description of the cDNA microarray platform can be viewed at GEO via accession ID GPL3608.

### Microarray experiment

The microarray experiment was carried out using a two-channel common reference design with two independent RNA extractions from each tissue sample. For each total-RNA extraction, 20 µg was labeled with Alexa 594 and 20 µg with Alexa 488 using SuperScript Indirect cDNA labeling System from Invitrogen. The RNA extracts labeled with Alexa 488 was collected from all tissue samples, mixed and used as reference sample referred to here as a the common reference. The RNA extracts labeled with Alexa 594 were used individually as labeled tissue samples. The labeled RNA extracts were used in two batches of 23 hybridizations corresponding to the two independent RNA extraction batches. One labeled tissue sample RNA (Alexa 594) and one labeled common reference sample RNA (Alexa 488) was thus hybridized to each of 46 microarrays. The same common reference sample was thus used in all the 46 (2×23) hybridizations. Two rounds of hybridizations on a Discovery XT hybridization station from Ventana were carried out corresponding to the two RNA batches. The hybridized microarrays were scanned and converted into TIFF images using Scanner and ScanArray Express software from Perkin Elmer.

### Data processing and analysis

Spot detection and spot intensity quantification was done using GenePix Pro version 6.0 software from Molecular Devices. Processing of the data and computation of the gene expression ratios between the tissue samples and the common reference was carried out in the R statistical programming environment version 2.3.0 [http://www.r-project.org/] using various Bioconductor packages [30]. Expression ratios were defined by the signal intensity of channel one (tissue sample), divided by the signal intensity of channel two (common reference sample). The expression ratios were normalized without background correction using the print tip loess method [31] of the limma package [32] from Bioconductor. The entire data set from the microarray gene expression experiment set has been submitted to GEO [33] and can be queried via accession ID GSE4918. To identify cDNAs representing genes with significant differences between expression in a given tissue and the overall expression, defined by the expression in the other 22 tissues, we subjected the normalized data to an empirical Bayes method and adjusted the P-values for multiple testing using the false discovery rate (“fdr”) method [32]. This test was carried out for all the 23 tissues. We used 5% significance level (P-values≤0.05) as a threshold and Log2(ratio) >0 to select cDNAs representing positively regulated genes. GO BP term enrichment tests were carried out using the GOHyperG function of the GOStats library from Bioconductor [30] and the global testing of genes grouped by KEGG pathway was done using Bioconductor's globaltest package [34]. Only GO terms and KEGG pathways represented by 50 or more microarray cDNAs were included. Visualization plots and hierarchical clustering was done using SpotFire software version 8.2.1 with package DecisionSite for Functional Genomics. Additional porcine genome sequence representations for uncharacterized microarray genes were retrieved from Emsembl Trace Server [35]. For further analysis of the porcine cDNA and genomic sequences we used NCBI's TBLASTX web program to compare amino acid translated sequences with NCBI's nr sequence database [36] and the SMART web program [37] to predict protein domains in the translated sequences. To compare the porcine gene expression profiles with corresponding gene expression in homologous tissues of human we identified a similar experiment from human, which also used a cDNA-based microarray platform and a common reference design [7]. The data set from this experiment was downloaded at GEO using Accession ID GSE2193. Using the best reciprocal BLASTN [http://www.timelogic.com/] similarity hit with P-values at or below 10^−8^ between the microarray cDNA sequences in the two experiments and requiring that the cDNA sequences for orthologous genes be mapped to the same gene ID in the two experiments, we identified 3.861 orthologous gene pairs. For each of the 3.861 orthologous genes we randomly picked one cDNA per gene in both experiment. By tissue sample comparison we were able to identify nine common tissues represented in both experiments (Frontal cortex, heart, kidney, liver, lung, pancreas, spleen, stomach and thyroid). We used relative abundance (RA) values as a measurement for gene expression levels, a previously proposed approach for optimal cross-experiment comparability [38]. RA is defined by the raw signal intensity in each individual tissue divided by the total signal intensity in all nine tissues. A data matrix with 3.861 gene rows, 2×9 = 18 tissue columns and average RA values was created. For comparative analysis we applied computation of Pearson's correlation coefficients between common tissues across pig and human on the RA data matrix using the R statistical programming environment [http://www.r-project.org/].
